# Liraglutide Ameliorates β-Amyloid Deposits and Secondary Damage in the Ipsilateral Thalamus and Sensory Deficits After Focal Cerebral Infarction in Rats

**DOI:** 10.3389/fnins.2018.00962

**Published:** 2018-12-17

**Authors:** Hui-Li Zhu, Zhang-Pei Liu, Wan-Yong Yang, Da-Wei Dong, Ying Zhao, Bing Yang, Li-An Huang, Yu-Sheng Zhang, An-Ding Xu

**Affiliations:** ^1^Department of Neurology and Stroke Center, The First Affiliated Hospital, Jinan University, Guangzhou, China; ^2^Clinical Neuroscience Institute, Jinan University, Guangzhou, China; ^3^Department of Neurology, Stroke Center, Zhuhai Hospital Affiliated with Jinan University, Zhuhai, China

**Keywords:** cerebral infarction, glucagon-like peptide-1, liraglutide, β-amyloid, secondary thalamus damage

## Abstract

Focal cerebral infarction causes β-amyloid (Aβ) deposition and secondary neuronal degeneration in the ipsilateral thalamus. Thalamus is the subcortical center of sensory, the damage of thalamus could cause sensory deficits. The present study aimed to investigate the protective effects of liraglutide, a long-acting glucagon-like peptide-1 (GLP)-1 receptor agonist, on Aβ deposits and secondary damage in the ipsilateral thalamus after focal cerebral infarction. In addition, this study was conducted to investigate whether liraglutide could improve sensory function after focal cerebral infarction. Forty-two male Sprague–Dawley rats were subjected to distal middle cerebral artery occlusion (MCAO) and then randomly divided into liraglutide and vehicle groups, and 14 sham-operated rats as control. At 1 h after MCAO, rats in the liraglutide and vehicle groups were subcutaneously injected with liraglutide (100 μg/kg/d) and isopyknic vehicle, respectively, once a day for 7 days. Sensory function and secondary thalamic damage were assessed using adhesive-removal test and Nissl staining and immunostaining, respectively, at 7 days after MCAO. Terminal deoxynucleotidyl transferase 2’-deoxyuridine 5’-triphosphate nick end labeling and Western blot were used to detect neuronal apoptosis. The results showed that liraglutide improved sensory deficit compared to the controls. Liraglutide treatment significantly reduced Aβ deposition compared with the vehicle treatment. Liraglutide treatment decreased the neuronal loss, astroglial and microglial activation, and apoptosis compared with the vehicle treatment. Liraglutide significantly down-regulated the expression of Bcl-2 and up-regulated that of Bax in the ipsilateral thalamus compared with the vehicle group. These results suggest that liraglutide ameliorates the deposition of Aβ and secondary damage in the ipsilateral thalamus, potentially contributing to improve sensory deficit after focal cerebral infarction.

## Introduction

Focal cerebral infarction after distal middle cerebral artery occlusion (MCAO) not only causes the primary cortical infarction, but also results in secondary damage in the regions that have synaptic connections with the primary ischemic lesion (Zhang et al., 2012). The most common secondary damage is ipsilateral thalamic degeneration after cerebral infarction, including neuronal loss, gliosis, axonal degeneration, hyperphosphorylation of Tau protein, and β-amyloid (Aβ) deposits (Dong et al., 2014; Xing et al., 2014). Such thalamic damage leads to delayed neuronal recovery (Baumgartner et al., 2018). Thus, protection against secondary neurodegeneration after ischemic stroke would be of significant clinical value.

Glucagon-like peptide-1 (GLP-1) is a peptide with incretin activity that potently stimulates glucose-dependent insulin secretion from small intestinal L cells, in response to food ingestion (Holz et al., 1993). The GLP-1 receptor (GLP-1R) agonists liraglutide, exendin-4, and lixisenatide are approved for the treatment of T2DM, which do not affect blood glucose levels in normal glycemic subjects and can be administered to nondiabetic patients (Drucker, 2018). Moreover, GLP-1 is produced in the central nervous system (CNS), primarily in the brain stem. GLP-1R is widely distributed throughout the brain, including the thalamus, hypothalamus, hippocampus, brainstem, and cortex (Bullock et al., 1996). Currently, the GLP-1R agonists have been found to exert neuroprotective effects against neurodegeneration and cerebral ischemic injury (Kuroki et al., 2016; Batista et al., 2018; Kabel et al., 2018).

Inflammation is one of the common mechanisms of type 2 diabetes mellitus (T2DM), ischemic stroke and AD (Morgese et al., 2017; Yildirim Simsir et al., 2018). Chronic stress can activate neuroinflammatory pathway as well as microglial, which increasing Aβ deposition. Accumulation of Aβ can ultimately lead to the development of Alzheimer’s disease, depression and diabetes. Numerous studies show that GLP-1 analogs suppressed the expression of inflammatory genes such as NFκB1(p105), NFκB2(p100), p65, TNF receptor superfamily member 1A, and receptor-interacting serine/threonine kinase 2, subsequently inhibited the inflammatory factors expression, such as TNF-α, IL-1β, IL-6, IL-17, p65, and CD-163 (Huang et al., 2015; Wicinski et al., 2018; Zhang et al., 2018).

Liraglutide is a long-acting and acylated GLP-1 analog administered once daily, which noncovalently associates with albumin and shares 97% sequence homology with the human endogenous GLP-1. The maximum serum concentration be reached at 8–12 h after subcutaneous injection. The mean clearance rate of liraglutide was about 1.2 L/h and half-life was about 13 h, which made it suitable for administration once a day. Liraglutide can move across the blood–brain barrier (Hunter and Holscher, 2012). Our recent findings indicated that liraglutide improved the neurological deficits and reduced the infarct volume by blocking neuronal apoptosis and reducing excessive oxidative stress in a rat model of MCAO (Zhu et al., 2016). Recent studies have demonstrated that liraglutide reduced the Aβ plaque count in the cortex, halved the activated microglia numbers, and increased the number of young neurons in a mouse model of Alzheimer’s disease (AD) (Hansen et al., 2015).

The targets of the protective effects of GLP-1 analog are similar to that of the secondary damage after cerebral infarction. However, the potential effects of liraglutide against secondary damage following ischemic stroke are still unclear. This study aimed to investigate that the effects of liraglutide on Aβ deposits, apoptosis and secondary damage in the ipsilateral thalamus after focal cerebral infarction in rats. In addition, this study was conducted to investigate whether liraglutide could improve sensory deficit after focal cerebral infarction. This study aimed to investigate that the effects of liraglutide on secondary damage in the ipsilateral thalamus and sensory deficit after focal cerebral infarction in rats.

## Materials and Methods

### Animals and the Experiment Groups

The animal experimental protocols were approved by the Animal Care and Use Committee of Jinan University. All experimental protocols involving rats were approved by the ethical committee of Jinan University and performed in accordance with approved guidelines and regulations. A total of 48 males Sprague-Dawley rats (3 months old, 300–350 g) obtained from the Animal Experiment Center of Southern Medical University, Guangzhou, China. All rats were bred at SPF level environment in animal laboratory center of Jinan University. The rats were kept in the cage of polycarbonate, and high pressure sterilized corncob was used as padding in the bottom of the cage. The temperature was controlled at about 20–25°C and the relative humidity was 40–70%. The light cycle was 12 h, which was a normal circadian rhythm. Food and water were available at libitum. Thirty-four rats were subjected to distal MCAO and 14 rats as sham-operated control randomly. Focal cerebral cortical infarction was induced by distal MCAO as previously described (Bederson et al., 1986). In brief, the rats were anesthetized with 10% chloral hydrate (3 ml/kg, intraperitoneally). Under the operating microscope, the distal right middle cerebral artery (MCA) was exposed through a burr hole and occluded by bipolar electrocoagulation distal to the origin of the striatal branches, which caused permanent focal infarction in the right cerebral cortex. Sham-operated animals underwent the same surgical procedures except for electrocoagulation of the MCA. Body temperature was maintained at 37 ± 0.5°C by a heating pad during the whole surgical procedures and recovery periods. Twenty-eight rats with successful MCAO surgery were randomly assigned into liraglutide or vehicle group (14 rats per group), and the remaining six rats with failed MCAO were excluded from the experiment. Sham-operated animals underwent the same surgical procedures except for the electrocoagulation of the distal middle cerebral artery (14 rats).

### Liraglutide Treatment

At 1 h after distal MCAO, rats in the liraglutide and vehicle groups were subcutaneously injected with liraglutide (100 μg/kg/d, Novo Nordisk, Denmark) and isopyknic vehicle, respectively, once a day for 7 days. The sham-operated rats did not receive any injection.

### Adhesive Removal Test

Adhesive removal test was carried out blindly in all experimental rats to assess the sensory deficit as previously described (Schallert et al., 1983) at 7 days after MCAO. At first, the rats were familiarized to the testing environment, and then two small pieces of adhesive paper dots (of equal size, 0.4^∗^1 cm) were adhered to the distal-radial region on the wrist of each forelimb. The time to remove each stimulus from the forelimbs was recorded in three trials per day for each forepaw. Individual trials were separated by at least 5 min. After 3 consecutive days training, all the rats were able to remove the adhesive paper dots within 10 s and then subjected to distal MCAO. At 7 days after distal MCAO, the time to remove each stimulus from the forelimbs was recorded in three trials per day for each forepaw, the max observation time was 2 min.

### Tissue Preparation and Nissl Staining

At 7 days after distal MCAO, after a deep anesthesia with 10% chloral hydrate intraperitoneally, eight rats selected randomly from each group were perfused with 100 ml saline and subsequently with 250 ml 4% paraformaldehyde in phosphate buffer (0.1 mol/L, pH = 7.4). Brains were removed and placed into 4% paraformaldehyde for 3 days at 4°C. Then, the brain tissues were embedded in paraffin and sectioned coronally at 10 μm thicknesses with a paraffin slicer. Nissl staining was performed as follows: sections were dehydrated with ethanol and treated with xylene, followed by incubation with 1% cresyl violet (Sigma, United States) solution for 5 min at 50°C. They were then mounted after dehydration with ethanol.

### Immunohistochemistry

Immunohistochemistry was performed as follows. In brief, sections were pretreated for 10 min with hot (85°C) 0.01 mol/L citrate buffer (pH = 6.0), rinsed in phosphate-buffered saline three times for 5 min each, then treated with 3% hydrogen peroxide for 10 min followed by rinsing in phosphate-buffered saline three times. After blocking with 5% normal goat serum for 1 h at room temperature, the sections were incubated with the primary antibody for rabbit polyclonal anti-rodent Aβ3-16 (1:1000, #SIG-39151, Covance, Emeryville, CA, United States) overnight at 4°C. The sections were then rinsed in phosphate-buffered saline three times and incubated with peroxidase-marked mouse secondary antibody for 1 h. The signal was visualized with 3,3-diaminobenzidinetetrahydrochloride.

### Immunofluorescent Labeling

Sections were rinsed in phosphate-buffered saline (PBS, pH 7.4) and blocked with 5% normal goat serum for 1 h at room temperature. Then, the sections were incubated with the primary antibodies of NeuN, glial fibrillary acidic protein (GFAP) and Iba-1 (Cell Signaling Technology, United States) at 4°C overnight and corresponding secondary antibodies were applied for 1 h at room temperature, followed by counterstaining with 4,6-diamidino-2-phenylindole dihydrochloride (DAPI).

### TUNEL Staining

The fragmentation of genomic DNA was detected by *in situ* staining of DNA ends with TUNEL according to the manufacturer’s instructions (Roche, Germany). Briefly, the sections was incubated with 100 μl the terminal deoxy-transferase reaction mixture for 1 h at room temperature in the dark. Fluorescence signals were detected under a fluorescence microscope.

### Western Blot

iated and mature brain astrocytes The remaining six rats in each group were killed to measure the levels of Bcl-2 and Bax proteins in the thalamus by Western blot analysis. Under deep anesthesia, the rats were perfused intracardially with 50 ml ice-cold 0.9% saline. The right thalamus was immediately isolated on ice and homogenized in cell lysis buffer. The protein samples were separated on a 12% sodium dodecyl sulfate (SDS)/polyacrylamide gel and transferred onto polyvinylidene fluoride membranes (Millipore, United States). The membranes were incubated with Bcl-2, Bax, and glyceraldehyde phosphate dehydrogenase (GAPDH) antibodies (Cell Signaling Technology, United States) at 4°C overnight. Immunoreactive bands were visualized by increased chemiluminescence (Millipore, United States) using corresponding horseradish peroxidase-conjugated IgG secondary antibodies (Cell Signaling Technology, United States). The images were captured by the gel imager and quantified using Quantity One software (Bio-Rad, United States).

### Image Analysis and Quantification

Histological sections were examined and analyzed by one author who was not aware of the experimental protocol. Images were captured under a microscope equipped digital camera and analyzed with Image-Pro plus (IPP) image analysis software (Media Cybernetics, MD, United States). For Nissl staining and immunostaining, serial sections between Bregma 2.4 and 4.4 mm were selected at intervals of every sixth sections from each rat for quantification. The number of intact neurons with intact membranes and nuclei detected by Nissl staining cells in the thalamus was counted using IPP image analysis software within three nonoverlapping fields under 400 magnification (425^∗^320 μm^2^), and was quantified as the average neuron number per field on each section (Ling et al., 2009; Zhang et al., 2011). Meanwhile, for quantification of immunostained sections, the number of NeuN^+^, Iba-1^+^, or TUNEL^+^ cells within the ipsilateral thalamus was counted in the same way, and was expressed as the average number of NeuN^+^, Iba-1^+^, or TUNEL^+^ cells per field on each section. The immunoreactivity of Aβ and GFAP were measured on a single photo using IPP software. The immunoreactivity of Aβ was measured on a single photo using IPP software as previous described (Chen et al., 1999; Wang-Tilz et al., 2006). Briefly, the image of whole thalamus sections were obtained with a microscope equipped digital camera. A series of 10 random images on several sections was taken for each immunostained parameter to obtain a mean value for statistical comparison. Staining was defined via color intensity, and a color mask was made. The intensity of the contralateral thalamus was taken as color mask. The mask was applied equally to all images, and measurements were obtained. The intensity of the labeling was determined by the computer program and gave a gray value ranging from zero (black) to 256 (white). Immunohistochemical parameters assessed in the area detected include mean stained area, mean intensity of stain, and mean integrated optical density (IOD). The immunoreactivity of Aβ was appeared as diffuse small dots, and the mean IOD indicates the total amount of Aβ deposition.

### Statistical Analysis

All data were analyzed by SPSS 13.0 for windows (Abacus concepts Inc., Chicago, IL, United States). Data are expressed as the mean ± SEM. The date were analyzed using one-way analysis of variance followed by Bonferroni test for multiple comparisons. The correlation coefficients between the thalamic Aβ burden and sensory deficit was calculated by Pearson’s correlation coefficients. Differences were considered statistically significant when *P* < 0.05.

## Results

### Liraglutide Decreased the Mean Time of Adhesive-Removal at Affected Forepaw in Rats

**FIGURE 1:**
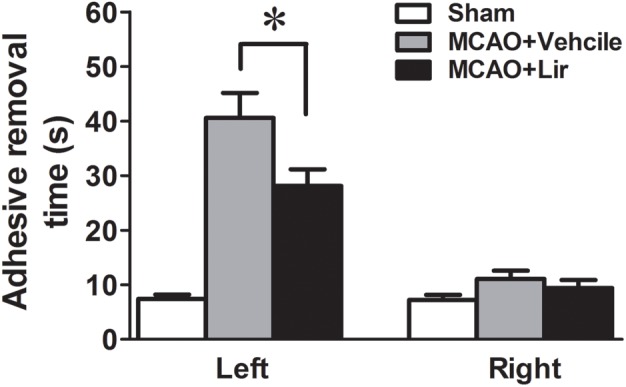
The adhesive removal test of rats treated with Liraglutide (*n* = 8), vehicle (*n* = 8) and sham-operated (*n* = 8) at 7 days after MCAO. ^∗^*P* < 0.05 vs. the vehicle group.

At 7 days after MCAO, the mean time to remove the stimulus from the left forepaw (affected side) increased significantly in liraglutide and vehicle groups compared with the control group (28.1 ± 3.1 and 40.6 ± 4.6 vs. 7.4 ± 0.8 seconds, all *P* < 0.001). However, liraglutide decreased the mean time of adhesive-removal at left forepaw compared with the vehicle group (28.1 ± 3.1 vs. 40.6 ± 4.6 s, *P* = 0.042). The time of adhesive-removal at right forepaw was no obvious difference among the three groups (all *P* > 0.05) (Figure 1). These results suggested that liraglutide improved the sensory impairment after focal cerebral infarction.

### Liraglutide Reduced Aβ Deposition in the Ipsilateral Thalamus After Focal Cortical Infarction

**FIGURE 2:**
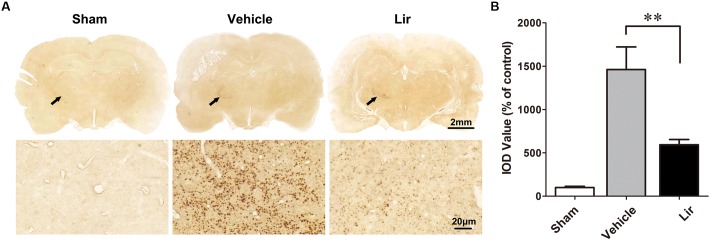
Aβ accumulation in the ipsilateral thalamus in sham-operated (*n* = 8), vehicle (*n* = 8), and liraglutide (*n* = 8) groups at 7 days after MCAO in rats. **(A)** Immumohistochemical staining of brain sections (10 μm) with anti-Aβ3-16 antibodies (arrows indicate the ipsilateral thalamus). **(B)** Quantitative analysis of Aβ deposits. ^∗∗^*P* < 0.05 *vs.* the vehicle group.

Compared with the sham-operated rats, strong Aβ immunoreactivity was revealed in the ipsilateral thalamus at 7 days after MCAO (Figure 2A). Aβ expression was localized as diffuse small dots (Figure 2A). Immunohistochemistry revealed that liraglutide treatment significantly reduced aggregated Aβ levels compared with the vehicle treatment (626.3 ± 121.4% vs. 1404.2 ± 216.0% of control, *P* = 0.028) (Figure 2B). The ipsilateral thalamic Aβ burden was reduced by 55.4% in the liraglutide group compared with the vehicle group.

### Liraglutide Increased the Number of Intact Neurons, Inhibited the Activation and Proliferation of Astrocyte and Microglia in the Ipsilateral Thalamus

**FIGURE 3:**
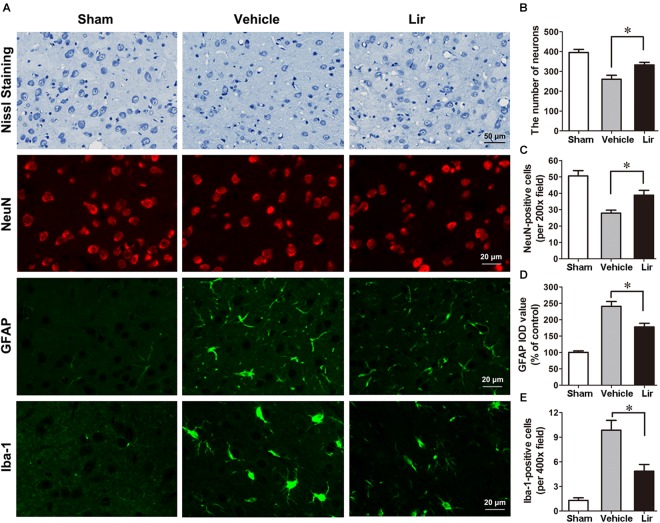
Nissl staining and immunostaining of rat brain sections (10 μm) in sham (*n* = 8), vehicle (*n* = 8), and Lir (*n* = 8) groups at 7 days after MCAO. **(A)** Representative photomicrographs of Nissl staining and immunofluorescent staining with NeuN, GFAP and Iba-1, respectively, in the ipsilateral thalamus. **(B)** Quantitative analysis of the number of intact neurons in the ipsilateral thalamus. **(C)** The quantification of NeuN-positive cells per 200× field in the ipsilateral thalamus. **(D)** Quantitative analysis of the IOD value of GPAP in the ipsilateral thalamus. **(E)** Quantitative analysis of the number of Iba-1 positive cells per 400× field in the ipsilateral thalamus. ^∗^*P* < 0.05 vs. the vehicle group.

Nissl staining showed that many abnormal neurons with shrunken cytoplasm and pyknotic nuclei are within the ipsilateral thalamus at 7 days after MCAO, but few in sham-operated group (Figure 3A). The number of intact neurons significantly decreased in the ipsilateral thalamic in vehicle and liraglutide groups compared with sham-operated group (*P* < 0.01 and *P* < 0.05, respectively) (Figures 3A,B). Compared with vehicle, liraglutide treatment significantly increased the number of intact neurons in the ipsilateral thalamic (332.8 ± 13.1 vs. 260.5 ± 19.9, *P* = 0.02) (Figure 3B).

In agreement with the findings of Nissl staining, NeuN-positive cells in the ipsilateral thalamic were reduced significantly in vehicle and liraglutide groups at 7 days after MCAO compared with sham-operated group (*P* < 0.001 and *P* < 0.05, respectively) (Figures 3A,C). However, the number of NeuN-positive cells in the ipsilateral thalamus was significantly increased in the liraglutide group than that in the vehicle group (39.0 ± 3.0 vs. 27.7 ± 1.7, *P* = 0.027) (Figure 3C).

The GFAP filaments are characteristic of differentiated and mature brain astrocytes. In the vehicle and liraglutide groups, the fluorescence intensity of GFAP in the ipsilateral thalamus was increased compared with the sham-operated group (both *P* < 0.001) (Figures 3A,D). Liraglutide treatment decreased the expression of GFAP compared with the vehicle group (177.7 ± 8.8 vs. 243.0 ± 15.0% of control, *P* = 0.012) (Figure 3D). The IOD value of GPAP was reduced by 26.9% in the liraglutide group compared with the vehicle group.

The number of ionized calcium binding adaptor molecule-1 (Iba-1)-positive microglias in the ipsilateral thalamus of the vehicle and liraglutide group were obviously increased compared with the sham-operated group (all *P* < 0.05) (Figures 3A,E). Liraglutide treatment significantly decreased the number of Iba-1-positive cells compared with the vehicle group (4.9 ± 0.7 vs. 9.8 ± 1.3, *P* = 0.021) (Figure 3E). These results indicated that liraglutide improved the secondary damage in the ipsilateral thalamus after focal cerebral infarction.

### Liraglutide Decreased Apoptosis in the Ipsilateral Thalamus

**FIGURE 4:**
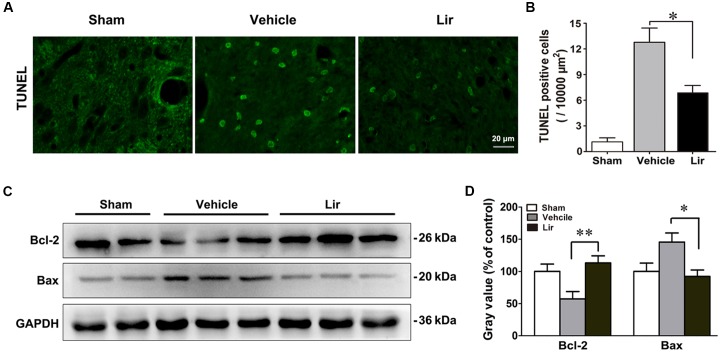
Liraglutide reduced apoptosis in the ipsilateral thalamus at 7 days after cerebral cortical infarction. **(A)** TUNEL staining of rat brain sections (10 μm) in sham (*n* = 8), vehicle (*n* = 8), and Lir (*n* = 8) groups. **(B)** Quantitative analysis of TUNEL-positive cells in the ipsilateral thalamus. **(C,D)** Western blot analysis of the expression of Bcl-2 and Bax in the ipsilateral thalamus in sham (*n* = 6), vehicle (*n* = 6), and Lir (*n* = 6) groups. ^∗^*P* < 0.05 and ^∗∗^*P* < 0.01 vs. the vehicle group.

Terminal deoxynucleotidyl transferase 2’-deoxyuridine 5’-triphosphate nick end labeling (TUNEL)-positive cells, representing the late stage apoptosis, were detected in the ipsilateral thalamus at 7 days after MCAO. Distal MCAO operation evidently increased the number of TUNEL-positive cells in the ipsilateral thalamus of the vehicle group compared with that of the sham-operated group (all *P* < 0.05) (Figures 4A,B). Compared with the vehicle, liraglutide significantly decreased the number of TUNEL-positive cells in the ipsilateral thalamus at 7 days after MCAO (6.7 ± 0.8 vs. 12.7 ± 1.8, *P* = 0.045) (Figure 4B).

The levels of Bcl-2 family of proteins, which are essential apoptotic regulators of mitochondria, were investigated using Western blot, in the ipsilateral thalamus at 7 days after MCAO. The Bcl-2 levels were significantly lower in the ipsilateral thalamus of the vehicle group than that of in the sham-operated group (*P* < 0.05) (Figures 4C,D). Compared with the vehicle, liraglutide increased the expression of Bcl-2 in the ipsilateral thalamus (113.2 ± 11.0% vs. 57.3 ± 11.4% of control, *P* = 0.006) (Figures 4C,D). On the contrary, the Bax levels in the ipsilateral thalamus of the vehicle group showed an increasing trend, although the difference with the sham-operated group was not statistically significant differences (144.9 ± 13.4 vs. 100.7 ± 14.9% of control, *P* = 0.092) (Figures 4C,D). However, compared with the vehicle, liraglutide significantly reduced the expression of Bax in the ipsilateral thalamus (89.9 ± 11.4 vs. 144.9 ± 13.4% of control, *P* = 0.027) (Figures 4C,D). These results confirmed the anti-apoptotic effects of liraglutide in the ipsilateral thalamus after focal cerebral infarction.

### Correlation Between Sensory Deficit and Thalamic Aβ Burden or Secondary Thalamic Damage

The mean time to remove the stimulus from the left forepaw at 7 days after MCAO was positively correlated with the thalamic Aβ burden and the number of TUNEL-positive cells (Table 1). Meanwhile, the mean time to remove the stimulus from the left forepaw was negatively correlated with the number of neurons in the ipsilateral thalamus detected by both Nissl staining and immunostaining for NeuN (Table 1). These results suggested that abnormal accumulation of Aβ and secondary damage in the ipsilateral thalamus associated with the sensory deficits after cerebral cortical infarction in rats.

**Table 1:** Pearson’s correlation coefficients between sensory deficit and thalamic Aβ burden or secondary thalamic damage at 7 days after MCAO.

	Vehicle (*n* = 8)		Lir (*n* = 8)		Pooled (*n* = 16)	
Removal time of left forepaw	*r*	*P*	*r*	*P*	*r*	*P*
Thalamic Aβ burden	0.789^∗^	0.020	0.769^∗^	0.026	0.843^∗∗∗^	<0.001
Number of TUNEL + cells in the ipsilateral thalamus	0.793^∗^	0.019	0.807^∗^	0.015	0.852^∗∗∗^	<0.001
Number of intact neurons in the ipsilateral thalamus with Nissl staining	−0.731^∗^	0.040	−0.766^∗^	0.027	−0.742^∗∗^	0.001
Number of NeuN + cells in the ipsilateral thalamus	−0.750^∗^	0.032	−0.757^∗^	0.030	−0.770^∗∗∗^	<0.001

*^∗^P < 0.05, ^∗∗^P < 0.01, ^∗∗∗^P < 0.001.*

## Discussion

Stroke is the second leading cause of long-term disability and death worldwide (Wang et al., 2017). Although many drugs have been demonstrated to have a neuroprotective ability based on laboratory results in ischemic stroke model, the vast majority of these candidate drugs have not been approved by clinical trials. Therefore, finding effective neuroprotective agents is of meaningful for acute ischemic stroke.

Thalamus is the subcortical center of sensory, the damage of thalamus could cause sensory deficits. Previous studies have reported that the secondary damage of thalamus caused sensory deficits after focal cerebral infarction in rats (Zhang et al., 2011; Xing et al., 2014). In the present study, we found that treatment with liraglutide reduced Aβ deposition, and ameliorated secondary damage in the ipsilateral thalamic in focal cerebral infarction rat model. Correlation analysis showed that abnormal accumulation of Aβ and secondary damage in the ipsilateral thalamus associated with the removal time of left forepaw after cerebral cortical infarction in rats. Therefore, the improvement of the mean time of adhesive-removal by liraglutide may be related to reducing the deposition of Aβ and secondary damage in the ipsilateral thalamic after focal cerebral infarction.

In our previous study, we found that N-[N-(3, 5-difluorophenacetyl)-L-alanyl]-S- phenylglycine t-butyl ester (DAPT), a functional γ-secretase inhibitor, treatment significantly attenuated the sensory impairments by reducing the secondary thalamic damage but without reducing the cortical infarct volume after MCAO in rats. The sensory deficit had a strong correlation with the secondary ipsilateral thalamic damage after MCAO in rats (Zhang et al., 2011). In our another previous study, treatment with liraglutide significantly reduced infarct volume in rats after focal cerebral infarction (Zhu et al., 2016). Therefore, liraglutide improved sensory deficit after focal cerebral infarction maybe associated with reducing the cortical infarct volume and ameliorating secondary damage in the ipsilateral thalamus.

Aβ, as a major component of senile plaque in the AD brain, is known to be neurotoxic. In general, Aβ monomers, oligomers and fibrils are all neurotoxic, although their toxicity may vary. In 2005, van Groen et al. (2005) firstly reported that the persistent presence and aggregation of Aβ precursor protein (APP) and Aβ, or their fragments, such as Aβ40, Aβ42, and Aβ3-16, to dense plaque-like deposits in the ipsilateral thalamus of rats after focal cerebral ischemia. Recently, a study has showed that Aβ oligomers, most notably the 30–40 and 50 kDa oligomers, were recognized to correlate with accelerated cognitive decline in rats of chronic stress exposure following photothrombotic stroke (Ong et al., 2017). In our previous study, we have found that reduction of thalamic Aβ3-16 by γ-secretase inhibitor significantly improved the sensory impairment and the secondary thalamic damage after MCAO in rats (Zhang et al., 2011). According to the previous results, we measured Aβ3-16 deposition in the ipsilateral thalamus after focal cerebral infarction in this study. The results of this present study suggested that liraglutide improved sensory deficit after focal cerebral infarction maybe associated with ameliorating the deposition of Aβ3-16 and secondary damage in the ipsilateral thalamus. However, whether liraglutide can reduce other types of Aβ need to be elucidated in greater detail.

Previous studies have shown abnormal Aβ deposits in the ipsilateral thalamus of rats after distal MCAO (van Groen et al., 2005; Ong et al., 2017). However, the mechanism of Aβ deposition in the regions away from the primary ischemic lesion is still unclear. Accumulation of Aβ in the cerebral centers involved in cognition and memory is a crucial aspect of the pathogenesis of AD (Hardy and Allsop, 1991). In 2003, Perry et al. (2003) first reported that a GLP-1R agonist reduced the levels of Aβ in the brains of mice and decreased the levels of amyloid precursor protein (APP) in cultured neuronal cells. Several studies confirm that some GLP-1R agonists decrease Aβ deposits in both *in vitro* and *in vivo* models of AD (Kosaraju et al., 2013; Wang et al., 2018). Glycogen synthase kinase (GSK)-3β may play an important role in the production of Aβ. The experiments has shown that inhibition of GSK-3β activity can block the production of Aβ peptides by interfering with APP cleavage at the γ-secretase step in a mouse model of AD (Chinchalongporn et al., 2018). Interestingly, GLP-1R agonists could decrease the activity of GSK-3β by activating the phosphoinositide 3-kinase/Akt pathway (Chen et al., 2016; Wang et al., 2018). Therefore, GLP-1R agonists may decrease the Aβ deposits by decreasing the activity of GSK-3β. In this study, we reported, for the first time, that liraglutide treatment significantly reduced Aβ deposits in the ipsilateral thalamus of rats after focal cortical infarction. Whether liraglutide could suppress the activation of GSK-3β by modulating the APP metabolism to hinder Aβ generation in the regions away from the primary lesion after cerebral infarction needs further elucidation.

There will be a series of inflammation after cerebral infarction, which contribute to active glial cell proliferation and increase the damage of neurons. In addition, the neuroinflammation are associated with Aβ toxicity. Mhillaj et al. (2018) has reported that soluble forms of amyloid-β (sAβ) neurotoxicity might be associated to COX-2-medid inflammatory pathways and early treatment with selective COX-2 inhibitor prevents the cognitive impairment of soluble Aβ-treated rats. Recently, many studies have shown that GLP-1R agonists have the effect on anti-inflammatory (Lee and Jun, 2016). Liraglutide treatment significantly reduced the inflammatory response in the cortex as measured by the number of activated microglia and prevented degenerative processes in the model of AD (McClean et al., 2011). In our present study, we also have found that liraglutide treatment significantly reduced the number of activated microglia in the ipsilateral thalamus after focal cerebral infarction. The glia may play a significant role in the CNS inflammation, and GLP-1 receptor was observed in astrocytes and microglia (Iwai et al., 2006; Gong et al., 2014). Iwai et al. (2006) has reported that GLP-1 prevented the LPS-induced IL-1 expression by increase of cAMP in astrocytes. In the present study, we have observed that liraglutide inhibited the activation and proliferation of astrocyte and microglia in the ipsilateral thalamus after cerebral infarction. However, we have not studied the mechanism in the present study, we will improve our research in the future work.

Small intestinal L cells secrete GLP-1 in response to food ingestion (Holz et al., 1993), which stimulates glucose-dependent insulin secretion in pancreatic β-cells. Studies in rodents and humans have illustrated that GLP-1R signaling plays a central role in the homeostasis of pancreatic β-cell mass function through stimulation of β-cell proliferation and inhibition of β-cell apoptosis (Kapodistria et al., 2018). Moreover, GLP-1 has extra-pancreatic effects, notably targeting the CNS to regulate appetite and satiety (Williams, 2014). Recent studies have found that GLP-1, as a neuropeptide, exerts neuroprotective, neurotrophic, and anti-inflammatory effects when released in the brain (Ji et al., 2016; Zhu et al., 2016; Gault and Holscher, 2018). Such studies have illuminated the anti-apoptotic effect of GLP-1 or GLP-1R agonist in the CNS. Bomba et al. (2018) found that a GLP-1R agonist inhibited apoptosis by decreasing p75 neurotrophin receptor-mediated signaling and promoted the activation of the brain-derived neurotrophic factor-tropomyosin receptor kinase B neurotrophic axis in mice. Another study showed that exendin-4, a GLP-1 analog, protected the brain by anti-apoptosis in type 2 diabetic rats (Candeias et al., 2018). Our previous study showed that liraglutide inhibited cell apoptosis by reducing excessive reactive oxygen species and improving the function of mitochondria in neurons under oxygen glucose deprivation (OGD) *in vitro* and MCAO *in vivo* (Zhu et al., 2016). There are two major apoptotic pathways: the mitochondrial and death receptor pathways. Bax and Bcl-2 play an important regulatory role in the mitochondrial apoptotic pathway. A previous study showed that Aβ deposits are accompanied by activation of caspase-3, down-regulation of Bcl-2, and up-regulation of Bax in the ipsilateral thalamus following cerebral infarction (Xing et al., 2014). In the current study, we found that liraglutide decreased apoptosis by regulating mitochondrial function in the ipsilateral thalamus of rats after focal cerebral infarction. The anti-apoptotic effect of liraglutide, mediated through a mitochondria-dependent pathway, is important for protecting neurons after cerebral ischemia injury.

Our previous study have showed that liraglutide reduced the infarct volume and improved the neurologic deficits in motor and somatosensory function in focal cerebral ischemic rat model. In the present study, we found that liraglutide ameliorates secondary damage in the ipsilateral thalamus and improves sensory impairment after focal cerebral infarction in rats. Therefore, liraglutide is likely to change the prognosis of acute ischemic stroke. At present, a clinical trail of treatment with GLP-1 analogs in acute ischemic stroke is being carried out (Muller et al., 2018). Our team are going to conduct a clinical trail on efficacy and safety study of liraglutide in acute ischemic stroke with T2DM.

## Conclusion

In summary, the present study reports, for the first time, that liraglutide prevents Aβ deposits and secondary damage in the ipsilateral thalamus, potentially contributing to improve sensory deficit after focal cerebral infarction. It may provide evidence for a novel therapeutic target against ischemic stroke.

## Author Contributions

H-LZ and Z-PL performed the experiments and wrote the manuscript. A-DX, H-LZ, and Y-SZ conceived and designed the study. Y-SZ and W-YY revised the manuscript. D-WD, YZ, BY, and L-AH reviewed and edited the manuscript. All authors read and approved this manuscript.

## Conflict of Interest Statement

The authors declare that the research was conducted in the absence of any commercial or financial relationships that could be construed as a potential conflict of interest.
